# Ordering global governance complexes: The evolution of the governance complex for international civil aviation

**DOI:** 10.1007/s11558-020-09411-z

**Published:** 2021-02-27

**Authors:** Mette Eilstrup-Sangiovanni

**Affiliations:** POLIS, University of Cambridge, Cambridge, UK

**Keywords:** Institutional Complexity, Regime Complexes, Global Governance, Complex Adaptive Systems, International Aviation

## Abstract

Many observers worry that growing numbers of international institutions with overlapping functions undermine governance effectiveness via duplication, inconsistency and conflict. Such pessimistic assessments may undervalue the mechanisms available to states and other political agents to reduce conflictual overlap and enhance inter-institutional synergy. Drawing on historical data I examine how states can mitigate conflict within Global Governance Complexes (GGCs) by dissolving or merging existing institutions or by re-configuring their mandates. I further explore how “order in complexity” can emerge through bottom-up processes of adaptation in lieu of state-led reform. My analysis supports three theoretical claims: (1) states frequently refashion governance complexes “top-down” in order to reduce conflictual overlap; (2) “top-down” restructuring and “bottom-up” adaptation present alternative mechanisms for ordering relations among component institutions of GGCs; (3) these twin mechanisms ensure that GGCs tend to (re)produce elements of order over time–albeit often temporarily. Rather than evolving towards ever-greater fragmentation and disorder, complex governance systems thus tend to fluctuate between greater or lesser integration and (dis)order.

International agreements and international law have expanded rapidly over the past century with the result that many areas of global politics are today co-governed by multiple institutions whose membership and competences overlap. The resulting “fragmentation” of global governance has been widely depicted as a source of rule inconsistency, duplication, and jurisdictional conflict (Abbott et al. 2015; Raustiala and Victor 2004; Alter and Meunier 2009; Kelley 2009:62; Orsini et al. 2013:32). Many worry that institutional overlap invites states to engage in regime shifting or forum shopping, thereby undermining the integrity of international law (Drezner 2009; Benvenisti and Downs 2007; Raustiala 2012; Morse and Keohane 2014; Orsini et al. 2013). Yet, others take a more optimistic view in arguing that inconsistency and conflict are not inescapable outcomes of growing institutional density, but that stable elements of coordination and collaboration can emerge within complex governance systems through overt management or gradual adaptation (inter alia, Forman and Segaar 2006; Oberthür and Stokke 2011; Gehring and Faude 2013, 2014; van Asselt and Zelli 2014; Henning 2017; Pratt 2018a).

This article builds on recent studies of inter-institutional coordination and collaboration to construct a theoretical account of how states and other political actors strive to order institutional complexes - and what factors condition their success. To explore processes of ordering, I adopt the concept of a Global Governance Complex (GGC), which I define as a system of diverse rule sets and agents that assert overlapping authority claims to govern a specific global policy issue. I have three main goals.

First, I seek to draw a conceptual distinction between “ordered” and “disordered” governance complexes according to the degree of synergy and coordination among separate component institutions.

Second, I theorize two mechanisms for ordering governance complexes through either “deliberate restructuring” or “spontaneous adaptation.” Deliberate restructuring entails the use by collective state principals of treaty-reform or the creation of cross-cutting institutions tasked with managing complexity. Spontenous adaptation refers to positive feedback processes in complex social systems which reinforce certain behaviours so that ongoing interactions among separate institutions tend—over time—to produce elements of order through mutual accommodation by individual institutions (Anderson 1999: 219). These mechanisms may be usefully conceived as “top-down” (or centralized) v. “bottom-up” (decentralized) ordering.

Third, I explain the prevalence of order versus disorder in GGCs as a function of institutional actors’ incentives and capacities for either top-down restructuring or bottom-up adaptation, which in turn reflect the alignment of state preferences and available organizational resources for adaptation.

The empirical part of the article examines processes of ordering in the GGC for international civil aviation from 1910 to 2010—as proof of concept.

I am not the first to observe that ongoing interactions among overlapping institutions can lead to stable divisions of labour through functional specialization or other forms of decentralized adaptation (see, inter alia*,* Aggarwal 1998; Oberthür and Stokke 2011; Gehring and Faude 2013, 2014; Pratt 2018a). My account expands on existing studies, however, by pointing to the frequency of deliberate, top-down restructuring of complex governance architectures through treaty terminations, mergers, reforms and funding cuts aimed to rationalize institutional interactions. I present “top-down restructuring” and “bottom-up adaptation” as alternative mechanisms for ordering GGCs and theorize under what conditions one or the other mechanism can be expected to come into play and to reverse institutional fragmentation.

In studying the evolution of order in GGCs this article extends the typical timeframe for studying institutional complexity to provide a longer historical backdrop. This move is animated by two concerns. First, many speak of institutional complexity as a *recent* phenomenon flowing from a present “erosion” of previously integrated, cohesive regimes (Raustiala and Victor 2004; Raustiala 2012:5; Betts 2013; Alter and Raustiala 2018:212; Pratt 2018a:564). However, the chief sources of institutional complexity—a steady increase in the number, breadth, and diversity of international institutions and agents—have been a persistent feature of many domains of international cooperation since the early twentieth century. As I discuss in later sections, international aviation governance was by most measures institutionally more complex during the first half of the twentieth century than it is today. By studying the evolution of global governance complexes in a historical perspective, we can hope to gain a better understanding of both the broad systemic conditions that fuel growing complexity, and of the proximate causes that trigger institutional proliferation in specific instances.

A second rationale for studying institutional complexity in a historical perspective is that most GGCs do not follow a linear path towards ever-greater complexity and fragmentation but rather evolve through stages of lesser or greater institutional density, overlap and (dis)order. A central claim of this article is that processes of top-down restructuring and bottom-up adaptation tend—over time—to (re)produce elements of both local and system-wide order in GGCs. Nevertheless, “order” in open complex systems remains precarious as existing task-divisions and exchange relationships are frequently upset by exogenous shocks (e.g. from the emergence of new actors or technologies, or new policy priorities). Thus, GGCs tend to evolve through a pattern of “punctuated equilibrium” (Krasner 1984; Colgan et al. 2012) in which periodic shocks lead to growing fragmentation and disorder, only to be followed by periods of (re)consolidation via deliberate management or spontaneous adaptation. A longer historical timeframe allows me to explore how processes of disintegration and reintegration unfold over time and to identify conditions leading to stability and change in complex governance systems.

The article is organized as follows. Section 1 introduces basic terms central to my discussion. The following section sketches my analytical framework. Section 3 applies this framework to international civil aviation governance since 1910—as proof of concept. I focus on international civil aviation for two reasons. First, governance of international civil aviation displays significant variation across time - both in terms of the number and diversity of institutions involved in policy-making and in the degree of ‘order’ in relationships among them. As a case study, civil aviation thus offers rich opportunity to observe causes of variation in patterns of complex governance over time. Second, international civil aviation governance has so far received relatively scant attention from international relations scholars.^1^ My analysis thus fills a gap in empirical knowledge.

My empirical analysis shows that both top-down restructuring and bottom-up adaptation are common mechanisms for ordering relationships among institutions. It also illustrates that order in the absence of centralized authority remains precarious in that existing exchange relationships are frequently upset by exogenous shocks which disrupt established patterns of interaction. Despite recurrent shocks, however, the GGC for civil aviation demonstrates a tendency to reestablish elements of order over time. Its evolution thus conforms to a cyclical pattern of punctuated equilibrium in which periodic shocks trigger growing conflict and disorder, only to be followed by periods of (re)consolidation and stability until a new crisis hits. In the concluding section I consider the implications of these findings for the wider study of institutional complexity in global governance.

## Definition of terms

The notion of a Global Governance Complex (GGC) builds closely on Raustiala and Victor’s (2004) concept of an international ‘regime-complex’ (RC) but differs in two respects. Victor and Raustiala define an RC as “an array of partially overlapping and non-hierarchical institutions governing a particular issue-area” (2004:278). This definition depicts the component parts of RCs simply as ‘institutions.’ Most applications of the concept, however, have taken “institutions” to imply intergovernmental treaties and organizations (but see Kahler 2020). For my purposes this is too narrow. Most areas of global politics are governed by a variety of institutions; formal and informal, state and non-state. Furthermore, global policy is (rather obviously) shaped both by *institutions as rules* (that is, ‘sets of rules, norms and decision-making procedures which guide actors’ expectations and behaviour’ Krasner 1982) and by *institutions as actors* (political agents engaged in formulating, implementing and interpretating relevant rules, such as international organizations, national government agencies, NGOs and multinational corporations). While fully compatible with the concept of a regime-complex, the notion of a global governance complex—a *system of diverse rule sets and agents that assert overlapping authority claims to govern a specific global policy issue*—highlights the diversity of rules (formal and informal) and agents (public and private) involved in global governance (see also Kahler 2020; Henning 2017).

A second contrast between GGCs and existing notions of regime complexes concerns the relationships between component rule-sets and agents. Raustiala and Victor cite the absence of hierarchy as a constitutive feature of an RC (2004:279. See also Alter and Meunier 2009:13; Alter and Raustiala 2018:184). But whilst a centralized hierarchical structure in the form of a single, dedicated umbrella IGO or centralized dispute resolution mechanism is clearly antithetical to the concept of governance complexity, informal hierarchy (or limited aspects of formal hierarchy) may exist in GGCs without negating their complex nature (Eilstrup-Sangiovanni and Westerwinter 2020; Henning and Pratt 2020). Furthermore, whereas many studies of regime complexes hold that the missions, principles and practices of overlapping institutions must be in conflict for an RC to exist (Orsini et al. 2013:32; Hale 2013; Alter and Raustiala 2018:184) conflict is not a necessary feature of a GGC. A central focus of this article is to understand the specific conditions in which inter-institutional conflict may be exacerbated or reduced. Rather than being constitutive of complexity, lack of hierarchy and conflict are thus treated as variable features that may differ across GGCs and across time.

A final question regards the boundaries of GGCs. While component institutions may address relatively clearly delimited issues (say, technical aspects of runway lighting), GGCs as a whole often sit at the nexus of several distinct issue-areas (Orsini et al. 2013: 30–1). For example, the GGC for international civil aviation resides at the intersection of international legal regimes for transport and travel and immigration, while also overlapping international agreements on public health and security. From this perspective, a GGC can be conceived as a system of separate but overlapping institutions and agents that together govern a particular issue but that may be interdependent with other institutions in adjacent governance systems. Like other forms of complex adaptive systems GGCs are therefore fundamentally “open systems” which interact in complex ways with the wider environments in which they are nested—importing and exporting “energy” from adjacent governance systems (Arthur 1999). It is largely this open nature which accounts for the tendency of GGCs to expand and contract over time, and to change their connective structures in response to changing environmental conditions.

## Ordering complex governance systems

In this section I consider what constitutes order in complex governance systems, *who* orders, and through what means. In doing so I draw on social system theory to disentangle notions of (dis)order from the notion of complexity.

In social systems theory, “complexity” refers to the number and diversity of elements that constitute a system and to the connections between them. The greater the number and diversity of elements and connections, the higher complexity (Anderson 1999; Schneider et al. 2017:186). Complex systems have distinct dynamic properties such as “non-linearity” (meaning that small changes may produce large, uneven effects) and “emergent behaviour” (entailing that system behaviour is irreducible to the behaviour of individual components but arises from interactions between them) (Anderson 1999; Kim and Mackey 2014). However, complexity is *not* synonymous with disorder or chaos. Simply put, “order” is an arrangement of things in relation to each other according to a particular, recognizable method or pattern. It refers to the nature of relations between a system’s components rather than their number or diversity. A governance system consisting of a handful of like institutions with limited ties between them will score low on complexity but may nevertheless be disordered if relationships are unpredictable and in constant flux. By contrast, a more complex system consisting of numerous diverse institutions with multiple ties between them may exhibit elements of order if institutional relationships are transparent and stable (Kim 2013; Kim and Mackey 2014).

### What does “order” in a complex governance system look like?

An early attempt at conceptualizing (dis)order in international governance complexes was made by Biermann et al. (2009:20) who distinguished “synergistic”, “cooperative” and “conflictive” fragmentation. Synergistic fragmentation refers to a governance system in which “the core institution includes (almost) all countries…and provides for effective and detailed general principles that regulate policies in distinct yet substantially integrated institutional arrangements.” Cooperative fragmentation describes a system in which “not all major countries participate in the core institution”. Conflictive fragmentation obtains when “institutions are barely connected”, or “when principles, norms and rules conflict.” (ibid.)

Distinguishing between “synergistic” v. “conflictual” relations among overlapping institutions provides a useful starting point for conceptualizing order in GGCs. However, by equating synergy with effective coordination through a “core institution” and conflict with “barely connected” institutions, the typology offered by Biermann and co-authors runs into conceptual difficulties by seemingly reproducing the dichotomy between “tightly integrated” *regimes* and “loosely integrated” *regime complexes* (Keohane and Victor 2011:8) - with synergy being a product of integration and conflict a product of fragmentation. It thereby risks conflating institutional complexity with conflict.

Rather than stipulate a measure of centralized coordination through an authoritative “core institution” my conception of order focuses directly on the degree of synergy v. conflict between component elements of a GGC. From this perspective, an “ordered” GGC is a governance system in which the rules and activities of separate institutions and agents offer complementary rather than conflicting policy instruments so that component institutions work—together or in parallel—towards solving joint problems (Ostrom 1990; Young 1994:110–11, 1996; Oberthür and Stokke 2011; Keohane and Victor 2011:8; Oberthür and Pozarowska 2013:104), and in which institutional exchange relationships are relatively stable. Such synergy may be achieved with or without coordination by a core institution to which most stakeholders belong. A “disordered” GGC, by contrast, is characterized by clashing agendas, incongruent governance procedures and unstable exchange relationships. Whereas institutions in an ordered complex are mutually reinforcing, disordered governance complexes have a zero-sum quality insofar as individual institutions detract from the ability of others to fulfil their goals.

What might ensure stable and synergistic relations among separate but overlapping institutions if not the existence of a coreinstitution that provides effective detailed principles for the complex as a whole (Biermann et al. 2009)? Drawing on social systems theory, I identify two sources of order in GGCs; task-differentiation and coordination or collaboration. Task-differentiation describes the extent to which institutions fulfil different functions or tasks (Child and Mansfield 1972). A high degree of task-differentiation reduces the potential for direct rule conflict or zero-sum competition for resources (Gehring and Faude 2013, 2014; Henning and Pratt 2020). Coordination refers to processes of information-sharing and mutual accommodation through which institutions harmonise their practices—formally or informally—while collaboration implies that institutions engage in exchange or pooling of resources. A high degree of collaboration reduces overall demand for resources and introduces scale-economies.

I leave aside “shared objectives” (Gehring and Faude 2013:124) or “domain consensus” (Skelcher and Sullivan 2008) as prerequisites of synergy in GGCs. Agreement on goals may reduce conflict over *what* gets delivered but not necessarily over *who* delivers - or on what terms. Shared objectives therefore do not per se lead to ordered GGCs but may coincide with fierce competition over resources and regulatory turf. In sum, “ordered” GGCs are characterized by task-differentiation and/or coordination or collaboration among component institutions leading to complementary governance practices and outcomes, whereas “disordered” GGCs are marked by inconsistent governance practices, duplication and turf-wars (Table 1). Importantly, I do not suggest that individual GGCs can be placed definitively in one category or the other. Far from being static, GGCs are constantly evolving systems, and—as a result—tend to move through stages of lesser and greater (dis)order.

**Table 1:** Ordered v. Disordered Governance Complexes

	Order	Disorder
Relations among component units	Synergistic = actions mutually reinforcing	Dysergistic = actions mutually undermining
Enabling/constraining mechanisms	• Task-Differentiation • Coordination and/or collaboration	• Undifferentiated Tasks • Lack of coordination or collaboration

Importantly, order is not synonymous with “effectiveness” understood as the ability to solve collective problems to the satisfaction of most stakeholders, let alone with “efficiency” (solving collective problems with minimum expenditure of time and resources). In a GGC featuring diverse institutions and agents, what amounts to effectiveness for one may represent failure for another. Thus, from the perspective of component institutions, the effectiveness of a GGC is largely in the eyes of the beholder. Effectiveness at the level of the system as a whole is equally difficult to measure: effective compared to what? Nevertheless, we may assume that conflicting rules and governance techniques which result in frequent contestation of established routines and exchange relationships (i) incur higher transaction costs, and (ii) are indicators that a system is failing to generate satisfactory outcomes in the eyes of many participants. Conversely, coordination of overlapping authority claims through mutual accommodation or overt collaboration are indicators of general “buy-in” to joint governance processes. Thus, an ordered GGC may be seen to signify general satisfaction with collective governance processes which may in turn be regarded as a proxy for effectiveness.

### Processes of ordering: Top-down v. bottom-up

Unlike their component institutions GGCs as a whole are not purposefully constituted but evolve through interactions among dispersed institutions and agents. This does not imply that institutional complexity is always unintentional. States may purposefully engineer institutional overlap to enable forum shopping (Raustiala and Victor 2004:301; Helfer 2009; Morse and Keohane 2014), or to reduce the clarity of legal obligations (Benvenisti and Downs 2007; Alter and Meunier 2009). International organizations may deliberately expand their activities into the domain of others in order to influence their behaviour or capture “market shares” (Raustiala and Victor 2004:301; Betts 2013). Yet institutional overlap may also arise “accidentally” because policymakers have limited knowledge of prior agreements entered into by their predecessors (Mallard 2014:448), or due to the rise of private governance schemes beyond the control of states (Abbott et al. 2015).

Whether deliberate or unintentional, institutional overlap may have undesired consequences. From the perspective of governments, regulatory overlap can result in significant added workloads and expenditure due to increased demands for national reporting and funding (Hofmann 2009; Keohane and Victor 2011). Inconsistent rules may result in conflicting practices which undermine collective goals. Institutional proliferation may also weaken state oversight. Multiple institutions with poorly separated mandates make it harder for states to keep track of how resources are allocated and used, or to place responsibility for poor results.

International organizations (whether public or private) may also suffer negative consequences from institutional complexity. Growing institutional density may trigger competition for scarce resources like state funding or expert staff. Jurisdictional overlap can also limit international organizations’ ability to induce policy change, as opportunities for forum shopping allow states and other target actors to evade commitments (Cooley and Ron 2002; Pratt 2018b). Thus, growing institutional density may generate demand for some form of management to reduce undesired effects. Governments, as institutional principals, may respond to such demand by taking purposive action to scale back overlap or reconcile conflicting rules through top-down treaty reform. However, order may also emerge from the bottom up as individual institutions adapt to competition through specialization, coordination or collaboration. I discuss each response in turn.

### Top-down restructuring

As the chief architects of international cooperation governments are well placed to manage institutional complexity by reengineering global governance architectures. Historically, governments have often dissolved existing international organizations or altered their mandates to eliminate undesired redundancy (Eilstrup-Sangiovanni 2018, 2020). An illustrative example is the Agency for International Trade Information and Co-Operation (AITIC) founded in 2004 to facilitate participation by Less Developed Countries (LDCs) in the global trading system. Soon after its founding concerns were raised that AITIC duplicated the work of existing bodies like the Advisory Centre on WTO Law, and the International Centre for Trade and Sustainable Development.^2^ In November 2008 several of AITIC’s founding members withdrew their membership citing “duplication” and “waste of funds”, and in 2010 AITIC was formally dissolved.^3^

Another tool for reducing undesired overlap is institutional fusion whereby governments combine existing institutions through merger or by formally subordinating one institution to another. An example is the 1987 merger of the anglophone West African Health Community and the francophone Coordination & Cooperation Organization for the Control of Major Endemic Diseases, which led to the creation of a new West African Health Organization (WAHO). According to ECOWAS member states, at whose behest the merger took place, “the driving force behind WAHO’s creation was the incongruence of the agendas that were being pursued by the two existing intergovernmental health organisations in the sub-region”.^4^

These brief examples are far from unique. Since 1900 hundreds of formal inter-governmental organizatoins (IGOs) have “disappeared” from the international system, being either dissolved or replaced by their founding states, merged with other organizations, or simply left to die (Eilstrup-Sangiovanni 2018, 2020). Informal intergovernmental institutions also frequently terminate (Vabulas and Snidal 2020). While these terminations may not all reflect a desire to reduce institutional overlap, they demonstrate that states habitually dispose of unwanted institutions. Even private IOs and NGOs are not immune to state-led restructuring as many depend on state funding which can be cut or made conditional on institutional change, or on enabling state regulations which can be changed.

In addition to showing governments’ capacity for top-down restructuring, historical data on institutional mergers and terminations illustrate a second point, namely that institutional overlap is not novel. As Fioretos (2020) shows, concern over loss of political control and costly duplication resulting from “the inordinate proliferation of world bodies” were chief reasons behind G7 leaders’ decision during a summit in 1975 to “resist further proliferation of global governance institutions” and to “…eliminate those [organizations] which do not have an essential purpose.” Looking still further back, the mandates and activities of many of the League of Nations’ functional agencies overlapped with those of pre-existing IGOs, especially in the fields of transport and health (Murphy 1994:83; Reinalda and Kille 2017:225). Likewise, the functions of post-war UN agencies governing agriculture, fisheries, telecommunication and trade often intersected the mandates of existing regional trade agreements. In some cases, rival authority claims were resolved by incorporating pre-existing institutions, first, into the League,^5^ and later into the expanding UN framework. In other cases, existing institutions saw their mandates altered to fashion a clearer division of labour. Finally, some redundant institutions were simply retired.^6^ In short, reducing institutional overlap through top-down reform is a longstanding practice.

A second mechanism whereby governments may manage complexity (in addition to institutional dissolution and reform) is to create cross-cutting institutions tasked with coordinating the activities of separate governing bodies. Previous studies have pointed to the creation of “super-institutions” tasked with promoting governance coherence (Forman and Segaar 2006:211; Oberthür and Stokke 2011:6). An example is the G20’s Financial Stability Board which synchronises the work of national and international financial authorities. More broadly, informal fora like the G7 and the G20 often serve to coordinate the agendas of formal IGOs (Fioretos 2020; Vabulas and Snidal 2020; Westerwinter et al. 2020).

Finally, top-down management of GGCs can also be forward-looking. As Reinsberg and Westerwinter (2021) show, founders of new international institutions often make design choices with an eye to avoiding conflictual overlap with incumbent institutions (also Raustiala and Victor 2004:280; Mallard 2014; Kim and Mackey 2014). For example, AITIC’s statutes explicitly directed the agency to “cooperate closely with related organisations” such as UNCTAD, The Advisory Centre on WTO Law and The International Centre for Trade and Sustainable Development with the aim “to coordinate activities and avoid duplication”.^7^ When undesired redundancy nevertheless ensued the agency was summarily dissolved.

As “masters of the treaty” governments are well positioned to (re)order relations among IGOs. However, states may also engage in restructuring of mixed public-private organizations, private for-profit IOs and NGOs via “orchestration” (Abbott and Snidal 2010; Abbott et al. 2015), through targeted funding cuts, or via market regulation.

### Bottom-up adaptation

A defining feature of complex adaptive systems (CAS) is that they exhibit “emergent behaviour”, meaning that undirected interactions between component elements generate distinctive behaviour at the level of the system as a whole. Starting in the 1980s, economists and organization scholars have drawn on theories of CAS in biology and physics to suggest that complex socio-economic systems may have capacity for spontaneous “self-organization” through “co*-*evolution” (Schneider et al. 2017). Simply put when repeated interaction among large numbers of separate elements involve positive feedback, some behaviours self-amplify and crowd out others. The result is that “starting in a random state, CAS tend to evolve toward order rather than disorder” (Anderson 1999: 219).

Leaning on such ideas, some observers have suggested that ongoing interactions between overlapping international institutions may gradually produce a stable division-of-labour as institutions adjust to one-another by developing increasingly separate tasks (Young 1994; Gehring and Faude 2013:214–15; Henning 2017; Pratt 2019:10). But while the notion of self-organization provides a useful trope, in the context of global governance this suggestion must be treated with caution. The concept of self-organization suggests that growing institutional density leads to increasing task-differentiation through a competitive (de)selection process whereby a given environment rewards institutions that perform certain functions or draw on certain resources and punishes others (Hannan and Freeman 1977; Halpin and Jordan 2009). Unlike biological systems in which individual elements have low capacity for short-term adaptation, however, GGCs comprise of bureaucratic agents that tend to actively seek to avoid failure by creatively adapting to their environments (Halpin and Jordan 2009:246–8; Gunitsky 2013:42; Abbott et al. 2016). It is thus incumbent on researchers to carefully identify the tools and incentives available to institutional agents for adaptation.

One bureaucratic response to growing institutional overlap might be to modify or reduce the scope of institutional activities to avoid direct competition with peers; a strategy of “avoidance” (see Bernholz 2009). Alternatively, weaker institutional agents may choose to defer to stronger peers by accepting their rules on issues where they are deemed more authoritative by virtue of having greater expertise or more powerful member states (Pratt 2018a). Individual institutions may also seek to specialize in functions or tasks that complement rather than compete with others. As Pratt (2018b) argues, task-differentiation both reduces direct competition for resources and shrinks the scope for regulatory arbitrage by states and other target actors since institutions perform distinct tasks which are not directly substitutable.

Bottom-up adaptation is not limited to ceding regulatory space but may also involve deliberate incursion into other institutions’ domains in order to capture a share of their resources (Skelcher and Sullivan 2008:758). Betts (2013) explores how growing competition from parallel institutions has led the UNHCR to expand its activities into areas in which it faces strong competition in an effort to recapture resources and pre-empt regime-shifting by states (see also Lesage and van de Graaf 2013). While the immediate effect of such task-expansion may be to increase regulatory conflict, insofar as other institutional agents choose to yield, the long-term result may be growing task-differentiation whereby individual institutions offer distinct but complementary solutions and depend on others to fill regulatory gaps (Abbott 2012).

As depicted thus far, bottom-up adaptation describes a fundamentally competitive process through which individual institutional actors seek to improve their fitness through either aggressive task-expansion, or by seeking out “niches” in which they can avoid direct competition (see Eilstrup-Sangiovanni 2019). An alternative strategy—highlighted by social exchange theory—is “cooperative adaptation” whereby institutions adapt to resource scarcity and hedge against risks by exchanging and pooling resources (Anderson 1999:186, 216; Skelcher and Sullivan 2008). Rather than simply reduce opportunities for conflict via differentiation, cooperative adaptation involves the creation of joint structures and processes by at least two organizations with the aim to increase mutual capacity to cope with environmental complexity (Schneider et al. 2017: 183).

Cooperative adaptation can be conceived as falling on a spectrum: At one end lies limited coordination of activities through regular information exchange. At the other end lies more far-reaching institutionalized collaboration through joint decision-making, sharing of staff, or co-financing of projects (Biermann and Koops 2017). Granting of mutual observer status falls towards the left end of the spectrum, while strategic industry alliances such as the Star Alliance among airlines and private standard-setting organizations fall towards the right end (Schneider et al. 2017:191). By building such formal alliances, institutions effectively “internalize” some of their environmental complexity.

The next section considers when GGCs may be subject to bottom-up v. top-down ordering. But first I must clarify two points. First, as already discussed, rather than being fully “spontaneous” bottom-up adaptation involves institutional agency and, as such, presupposes a degree of institutional autonomy. In the case of private IOs this assumption is relatively uncontentious. But in regard to state created IGOs one may wonder why, as bureaucratic agents*,* IGOs should have different incentives or capacities for ordering than their state patrons? I take it as uncontroversial that most IGOs are designed to operate with a degree of independence from member states in order to enable them to resolve cooperation problems. I further presume that most IGOs are staffed with state representatives and bureaucrats who—in addition to serving state interests—wish both to promote organizational objectives and keep their jobs. In short, IGOs serve member states’ interests but are not synonymous with member states. Thus, even when top-down reform is blocked due to conflicting state preferences, room for manoeuvre may exist for bottom-up adaptation by organizational agents.

Second, it is important to emphasize that while both task-differentiation and cooperative adaptation involve *deliberate* action by institutional agents to adapt to environmental pressure, the resulting system-wide order remains “undirected.” Consistent with bounded rationality assumptions, institutional agents in GGCs are presumed unable to forecast the system-wide consequences of their individual choices (Anderson 1999). Instead, each act to improve their own survival chances. Rather than evolving according to an overarching plan (as top-down restructuring may), bottom-up adaptation is thus an “emergent” dynamic feature of GGCs driven by the co-dependent choices of individual institutional agents each of whom seeks to adapt strategically to its local circumstances (Anderson 1999).

### Who orders, when?

When should we expect governments to attempt (and succeed) in ordering GGCs? When should we rather expect bottom-up adaptation? State incentives to order GGCs depend firstly on the nature of interdependencies between existing institutions. Whereas overlapping institutions may be mutually undermining due to incompatible rules or excessive transaction costs, co-governance by diverse actors may also increase flexibility and widen technical and political knowledge thereby reducing policy uncertainty and increasing problem-solving capacity (Keohane and Victor 2011; Abbott and Faude 2019). When overlapping institutions create positive spill-overs, states will have few incentives to engage in top-down restructuring. But when overlap results in costly duplication or weakened oversight, states can be expected to attempt to reduce negative effects (see Johnson and Urpelainen 2012 on negative spill-over as a spur to regime integration). Whether institutional overlap produces negative or positive spill-over is partly an empirical question. As a rule, however, the greater the number of institutions governing an issue, and the greater the duplication of tasks, the more likely that excessive governance costs will trigger demand for restructuring.

1. Demand for top-down restructuring increases with increasing institutional density and overlap.

Next to demand, top-down restructuring also depends on how easily existing institutions can be dislodged or modified; and at what cost (Fioretos 2017, 2020). Institutional principals are composite actors. If member states are united in their desire for change, reducing overlap through treaty-reform, merger, dissolution or funding cuts may be relatively unproblematic. But when preferences differ over which institutions to keep and which to reform (and how) barriers to restructuring mount.^8^ In such conditions, top-down restructuring may either fail altogether or be limited to the creation of cross-cutting coordinating institutions alongside existing designs, consistent with notions of “institutional layering” (Fioretos 2017:15–19, 2020; Hofmann 2013; Haftel and Hofmann 2019). In this case, ordering effectively goes hand-in-hand with institutional proliferation; the system is growing simultaneously more complex and more ordered.

Hypothesis 2 Successful top-down restructuring is most likely when states hold similar views on which institutions are essential and which are superfluous or counterproductive. In contrast clashing preferences may impede action or trigger further proliferation of cross-cutting institutions.

When should we expect bottom-up adaptation? The short answer is: In the absence of successful top-down restructuring. If successful, top-down interventions to reduce the number of institutions governing an issue or untangle their mandates will lessen pressure for bottom-up adaptation. However, bottom-up adaptation also abides by its own dynamics. From an ecological perspective, growing institutional density affects populations of institutions such as GGCs through two sequential processes: “mutuality” and “competition” (Hannan and Caroll 1992). Early in a population’s development, when density is relatively low, the fact that more institutions emerge with a specific functional focus or form tends to make that form more acceptable to key audiences through “joint legitimation” (Hannan and Caroll 1992:51; Abbott et al. 2016). At this stage, more institutions with similar focus may increase opportunities for mutually beneficial information-exchange and joint learning, while relative resource abundance^9^ means individual institutions can expand their tasks without coming into direct conflict with others. At this stage, individual task-expansion may thus go hand-in-hand with limited coordination.

Yet as institutional density grows (due to the emergence of new institutions and/or task-expansion by existing ones) resource scarcity may set in, constraining further expansion and fuelling competition (Hannan and Freeman 1977; Abbott et al. 2016:9). In such conditions, individual institutions come under pressure to adapt through either differentiation or cooperation. Adaptation through task-differentiation a priori entails fewer risks than cooperation insofar as institutions preserve strategic and operational independence. However, task-differentiation places high demands on organizational resources in order to expand or alter existing work portfolios, change internal routines, or develop new specialist skills. As resource scarcity grows, exchanging and pooling resources may therefore become essential to survival. At the same time, institutions with differentiated governance functions are also more likely to offer complementary services that lend themselves to collaboration (Abbott and Faude 2019). We should thus expect bottom-up adaptation to occur through a sequential pattern, starting with competitive task-differentiation and evolving through growing inter-institutional cooperation.

Hypothesis 3 Bottom-up adaptation increases as a function of institutional density in the absence of state-led restructuring.
Hypothesis 4 Bottom-up adaptation is expected to manifest, first, in task-differentiation and second, in cooperative measures like resource-exchange and alliance-building.

### Stability and change

My theoretical framework suggests that, as institutional density and overlap grows, processes of either top-down or bottom-up ordering will set in. Together these twin dynamics imply that—in stable environments—GGCs will tend to display growing elements of order insofar as institutional relationships grow less conflictual and more synergistic over time (Fioretos 2017; Oberthür and Stokke 2011; Gehring and Faude 2014). Global policy environments are of course rarely stable (Gehring and Faude 2014). The porous boundaries of most GGCs means they are often subject to exogenous shocks in the form of new actors or new technologies which lead to new governance tasks (Young 1994) or changing power balances which alter states’ preferences (Gehring and Faude 2014). Rather than moving towards ever-greater order, GGCs are therefore likely to evolve through a process of punctuated equilibrium in which periodic shocks upset existing exchange-relationships and lead to growing disorder, only to be followed by processes of (re)ordering which stabilize relations until a new shock hits.

Hypothesis 5 GGCs evolve according to a pattern of punctuated equilibrium where periodic shocks lead to growing disorder, followed by periods of re-ordering through top-down restructuring or bottom-up adaptation.

The next section examines to what extent the historical evolution of the GGC for civil aviation fits these expectations. While a single case study cannot provide a rigorous test of my hypotheses, it clearly demonstrates the relevance of my conceptual and theoretical framework. Furthermore, significant variation over time in the number and type of governance institutions in the aviation domain and in the relationships between them offers rich opportunity to observe the factors and processes leading to growing order or disorder. This variation is crucial for my case-selection. While I expect my model of top-town v. bottom-up ordering to apply to all GGGs, some governance complexes—for example, the GGC governing cybersecurity—are too young to display much variation and would thus offer limited empirical insights regarding the evolution of complexity. Other domains (say, nuclear non-proliferation) mainly comprise government-led institutions with low autonomy, leaving less scope for bottom-up adaptation. Undoubtedly, a comparative research-design featuring systematic comparison to other domains such as international trade or human rights would offer additional insights. Yet such a design would take me beyond the limits of a single article. Instead I focus on exploring within-case variation and process-tracing to demonstrate the plausibility of my theory.

Observationally, I expect exogenous shocks to lead to growing institutional density and resulting resource competition. These are my main independent variables at the system level. I then look for evidence of top-down restructuring and bottom-up adaptation. My framework predicts top-down restructuring when governments are relatively united in their desire for institutional change. Given a failure of top-down ordering, a more gradual process of bottom-up adaptation is expected; firstly via competitive task-differentiation and more gradually via growing cooperation. Importantly, the level of task-differentiation/cooperation is a property of the system as a whole, rather than of any single institution or pair of institutions. However, I assume that system-level changes are driven by underlying changes in the behavior of individual units. I therefore consider how individual institutions adapt to complexity through specialization or cooperation. The behavior of institutional agents is ultimately what is “ordered” through processes of adaptation.

## The global governance complex for international civil aviation

 Despite the profound cross-border implications of international civil aviation there has never been a single, integrated regime governing international civil aviation. Since the early 1900s international aviation has been regulated by an array of overlapping bilateral and multilateral agreements implemented by IGOs, NGOs, individual government agencies, and private commercial actors. The complexity of international aviation governance is partly dictated by the complexity of air travel itself. For international flights to operate smoothly, numerous legal regimes must co-function, including those governing radio and telecommunications, meteorological services, and customs. International aviation touches directly on major economic and social issue-areas such as commerce, tourism, and immigration. At the same time, the speed and reach of commercial flights give rise to overlap and potential jurisdictional conflict vis-à-vis international regimes governing public health, environmental protection, and security. As a result, decisions affecting international air travel are made in multiple venues, many of which do not focus directly on aviation.

The complexity of international aviation governance is, however, not merely a function of its technical aspects. Functions at the heart of aviation governance, such as the granting of overflight and landing rights, licensing of airlines, fixing of international routes and fares, and standardization of safety procedures are all made by multiple separate institutions with overlapping functions and membership. Despite periodic attempts to integrate these functions into a single regime, international aviation governance has remained strongly fragmented. The system is thus a complex governance system *par excellence.*

Since the early twentieth century, the GGC for international aviation has undergone several changes that can be described as fundamental restructurings of its institutional architecture. Along with examining the roots of complexity, this section focuses on how the system has evolved over time; expanding and contracting in terms of the number, diversity and density of component institutions, and alternating between periods of greater and lesser (dis)order.

### 1910–1943: Foundations of a complex governance system

The first international flight was accomplished in 1909 by French pilot, Louis Bleriot, crossing the Channel to England. The following year, France hosted the first international aviation conference to agree basic principles of international air law. This conference exposed a rift between opposing institutional designs. On one side stood the ideal of international “freedom of the air” supported by Germany and France; on the other, the model of sovereign control of national airspace, favoured by Britain (Jönsson 1981:276; Nayar 1995:146–47). National delegates failed to reconcile these clashing views and during the following years individual European governments passed legislation establishing their right to exclude foreign aircraft from their airspace (Jönsson 1981:277). The failure of intergovernmental cooperation meant that the first international institution to address civil aviation was instead a private organization; the International Legal Committee on Aviation (ICLA), founded in 1909 by an independent group of lawyers. Until 1930, the ILCA substituted for a lack of international regulation by drafting an advisory International Code for the Air summarizing articles of public and private air law.

Attempts at intergovernmental cooperation were renewed after World War I. Among the chief concerns of state representatives to the Paris Peace Conference in 1919 were military uses of civilian aircraft technology and the need to maintain strong communication and transport ties with overseas colonies (Jönsson 1981:278; Nayar 1995). The Conference agreed on a Convention Relating to the Regulation of Aerial Navigation (the “Paris Convention”) which addressed technical and operational aspects of civil aviation and created the International Commission for Air Navigation (ICAN) to oversee the development of technical standards. On the contentious issue of rights to commercial use of international airspace, however, no agreement could be reached. Article I of the Paris Convention confirmed that each state “enjoys complete and exclusive sovereignty over the airspace above its territory.” In practise this led to a highly decentralized system of cooperation whereby states had to negotiate with individual foreign governments for rights to overfly and land on their territory (Nayar 1995:147).

In addition to confirming the principle of unreserved “air-sovereignty”, the 1919 Paris Conference introduced a geographical divide in international aviation governance. The Paris Convention was ratified by the British Empire, seven European air-faring states (excluding Germany), and Japan. The United States and the Soviet Union declined to join, preferring to develop their own regional agreements (Jönsson 1981:277–78). Panama, Bolivia and Chile also signed the Paris Convention but soon denounced it in order to join the Pan-American Convention on Commercial Aviation (PACCA) which was negotiated in 1928 under US leadership. By 1928 cooperation on technical aspects of international aviation thus took place in two geographically separate fora—ICAN and PACCA—whereas commercial access to foreign airspace was governed by bilateral agreements.

Washington’s rejection of ICAN echoed wider US resistance to the League of Nations under whose direction ICAN was placed^10^ (Dierikx 1992). However, the geographical divide in aviation governance was also a reflection of technology; inter-continental commercial passenger flights were not yet feasible in 1919, limiting the need for a global agreement.^11^ Given rapid advances in technology, however, it soon became apparent that neither ICAN nor PACCA could alone resolve the many technical, political, economic and security questions arising from fast-expanding international air travel. Although there was no direct overlap in membership between the two institutions, their co-existence nonetheless introduced clear potential for rule-conflict insofar as each institution regulated issues which—given the increasingly global reach of civil aviation—had direct implications for the operation of the other. But whilst technological progress dictated closer coordination, or even integration, between ICAN and PACCA, the clashing preferences of powerful states whose capacity for long-haul aviation varied greatly meant that neither institution could be easily expanded to provide joint regulation under a single global regime.

During the 1920s, several international conferences were convened to negotiate a centralized global aviation regime. All ended in deadlock (ICAO-History). Instead, growing demand for regulation led to a rapid proliferation of task-specific bodies. During the 1920s and 1930s, a cascade of new institutions emerged alongside ICAN to address technical issues arising from expanding air-traffic; from the allocation of routes, to radio-communications, weather forecasts, and operation of signals and lights (ICAO-History). These institutions included standing international aviation conferences among West and Central European states and sub-regional conferences such as the Mediterranean Aeronautical Conference and the Aeronautical Conference of the Baltic States and the Balkans. Many existing IGOs like the International Office of Public Hygiene, the International Meteorological Organization, and the International Radiotelegraph Union also placed aeronautical issues on their agendas (ibid). A limited measure of information-exchange and coordination of technical standards was provided by the International Technical Committee of Experts in Air Law, founded in 1925, which counted members from both PACCA and ICAN (Ide 1932; Wilberforce 1947). All other attempts at top-down rationalization stumbled on opposing state preferences.

Alongside the proliferation of public institutions, numerous private bodies emerged to address aeronautical matters. The most prominent was the International Air Traffic Association (IATA) founded in 1919 by representatives of major airlines to harmonize international operations. Other private institutions included the non-profit Aircraft International Register (1926), the Aerospace Medical Association (1929), and the International Committee for the Study of Sanitary Aviation (1934). This growing assemblage of private institutions filled regulatory gaps left by states’ inability to agree a centralized regime to govern aviation safety and to allocate international routes and carrier capacities. Thus, overall, institutional density and overlap was rapidly growing (see Table 2) while conflicting state preferences meant that top-down ordering was stalled (Hypothesis 2).

**Table 2:** The Global Governance Complex for International Aviation, 1900–1944

Institutions dealing exclusively w. international aviation	Founding year	Nature
International Aeronautical Federation	1905	Private
International Legal Committee on Aviation	1909	Private
International Commission for Air Navigation (ICAN)	1919	Public
International Air Traffic Association (IATA)	1919	Private
International Technical Committee of Experts in Air Law	1925	Public
International Aircraft Register	1926	Private
Pan-American Convention on Commercial Aviation (PACCA)	1928	Public
Regional Aeronautical Conferences	–	Public
International Aeronautical Congresses	–	Public
Permanent Committee of International Congresses of Sanitary Aviation	1929	Private
Aerospace Medical Association	1929	Private
International Committee for the Study of Sanitary Aviation	1934	Private
Convention for the Unification of certain rules for Intl. carriage by air (1933)	1933	Public
**Institutions dealing secondarily with international aviation**		
International Radiotelegraph Union	1906	Public
Pan-American Union	1910	Public
League of Nation’s Committee on Transit and Communications	1919	Public
League of Red Cross Societies		
**Institutions with functions intersecting ICAN**		
International Conferences on Radio Communications	–	
International Meteorological Organization	1873	Public
International Law Association	1873	Private
Universal Postal Union	1874	Public
International Commission on Illumination	1900	Private
International Office of Public Hygiene	1907	Public
International Hydrographic Bureau	1921	Public

Adapted from ICAO: https://www.icao.int/secretariat/postalhistory/international_aviation_organizations_working_alongside_ican_part_1.htm?

#### Failure of top-down restructuring leading to bottom-up adaption

As institutional density and overlap grew so did demand for coordination (Hypothesis 1). Among the issues that could not be effectively addressed by any regional institution was the liabilities of private air carriers operating in international airspace. If an international airliner caused death or injury to passengers, or surface damage to third parties, who would be liable? How could one protect passengers’ rights while also shielding national airlines from crippling compensation claims from third parties? (Wilberforce 1947). Given ICAN’s and PACCA’s limited membership, it was decided that an international agreement on carrier liability could best be achieved independently of either body. In 1929, the International Technical Committee of Experts in Air Law drew up a Convention for the Unification of Certain Rules Relating to Carriage by Air (“Warsaw Convention”). Ratified by thirty-four states, including all the major airpowers, the Warsaw Convention became the first aviation convention to gain a global membership (Wilberforce 1947). However, its remit remained limited to financial liabilities of international air-carriers whose operation and safety procedures continued to be governed separately by PACCA and ICAN. The Convention thus illustrates how growing demand for coordination against a backdrop of divergent state preference can lead to further institutional proliferation rather than reform-based restructuring (Hypothesis 2).

As predicted, failure by governments to restructure an increasingly complex governance system was gradually compensated by bottom-up adaptation (Hypothesis 3). The parallel operation of a large number of overlapping aviation institutions increased operational costs due to complicated information-exchange and widespread duplication. Often, the same technical and legal experts were represented in different aviation institutions, doing parallel work.^12^ To reducecosts, individual institutions gradually developed procedures for coordinating activities. Given significant overlap in the mandates of ICAN and the League of Nation’s Committee on Transit and Communications, the two organizations were normally represented at each other’s meetings when issues of common concern were discussed (ICAN.Org). ICAN’s Director also attended the many regional aviation conferences (ICAN.org). Among private institutions, the International Committee for the Study of Sanitary Aviation created in 1934 introduced an element of coordination between the International Aeronautical Federation, The Permanent Committee of International Congresses of Sanitary Aviation, and The League of Red Cross Societies to develop joint guidelines for aviation health, and to liaise jointly with the public International Office for Public Hygiene.^13^

The fact that adaptation was achieved mainly through informal coordination rather than task-differentiation or more permanent institutionalized cooperation may be explained by a relatively resource rich environment. During this period more and more states developed capacities for international air transport. Continuous market-growth combined with rapid technological progress which required constant updates to existing protocols and procedures meant that individual institutions could expand their functions and membership without coming into direct conflict, while reducing governance costs and increasing effectiveness through decentralized coordination. Given relatively abundant resources, institutional relations thus developed in broadly synergistic ways with individual institutions supplying overlapping but mutually reinforcing tasks, and parallel processes of standard development facilitating communal learning through regular information-exchange.

### 1944–1972: Exogenous shock and eventual re-ordering

This harmonious state of affairs ended during World War II due to two exogenous shocks. First, the war effort led to the creation of new international air routes and bases—from New York to Paris, from Africa to the Arctic—unleashing an international race to expand commercial air-travel (Mackenzie 1993:107). Second, the war dramatically changed the balance of power among states as the US emerged as the leading global producer and operator of long-haul flights (Nayar 1995).^14^

As war drew to an end, the US Government invited the Allied powers to Chicago to discuss plans for postwar aviation (Mackenzie 1993:111). On the agenda was (i) universally recognized navigational signals and other technical standards; (ii) international rules for commercial air transport (Lowenfeld 1975:37). The first issue was easily settled, but not the second. Given its newfound competitive advantage, the US pushed for a minimalist “open skies” regime in which market forces would determine the routes and capacities of international carriers (Nayar 1995:146; Mackenzie 1993:108). Realizing that its technologically inferior aviation industry could not compete with the Americans in an unregulated market Britain pushed instead for a central regulatory body mandated to allocate routes, fix capacities, and determine fares on an “equitable basis” (UK White Paper 1944; Lissitzyn 1964; Dierikx 1992:807–8). To strengthen its negotiation position, Britain in November 1944 created the Commonwealth Air Transport Council to coordinate aviation policy among Commonwealth countries – an arrangement decried in Washington as an “empire-cartel” (Lissitzyn 1964).

Wartime correspondence shows deep tensions between US President Roosevelt and British Prime Minister Churchill over the issue of civil aviation. Churchill described Washington’s pro-market position as an attempt to “hijack the global skies” (Dierikx 1992; Mackenzie 1993:107–8). Roosevelt in turn warned that failure to accept US proposals might jeopardize the continuation of Lend-Lease (Schlesinger 1984). So deep ran the conflict that Roosevelt warmed Churchill in a Telegram in November 1944 that “Congress and the public will wonder about the chances of our two countries…working together to keep the peace if we cannot even get together on an aviation agreement”.^15^

In the end the Chicago Convention was a victory for America. Article 6 reaffirmed the principle of unabridged air sovereignty by stating that “no scheduled international service may be operated over or into the territory of a contracting State, except with the special permission [of that State].” ICAN was dissolved and replaced by the International Civil Aviation Organization (ICAO), which was tasked with ensuring “maximum uniformity in regulations and standards of civil aviation” by “recommending certain practices that member countries should follow” (Art. 44).^16^ Like ICAN before it, ICAO’s role would be limited to technical matters and be purely advisory. On the issue of commercial air rights, no agreement was reached. Instead the issue was covered in two supplementary agreements: The International Air Services Transit Agreement (which guarantees the freedom of civilian aircraft to overfly foreign territories), and the International Air Transport Agreement (granting freedom to transport passengers and cargo to/from one’s homeland and other countries). Neither agreement forms part of ICAO’s constitution, binding only states that have separately ratified them (Jönsson 1981:81). In short, despite hopes for the contrary, the Chicago conference became a basis of legal fragmentation.

Renewed failure to agree an integrated international aviation regime led to further institutional proliferation (Hypothesis 2). In the decades following the Chicago Conference more than 1000 bilateral accords were made exchanging commercial air rights - their content varying depending on the relative bargaining strength of the parties (Lissitzyn 1964:248). Meanwhile the frequency and capacity of international flights were determined by individual airline operators according to consumer demand. On the issue of fares some progress was made in 1946 when Britain and the US agreed at Bermuda to allow the private airline association, IATA, to centrally fix air fares, subject to government approval (Goodwin 1964; Lissitzyn 1964; Nayar 1995:158). This arrangement soon become a blueprint for other countries. A private body, IATA, thus stepped in to supply a governance function on which governments could not agree (Jönsson 1981).

#### Limited top-down restructuring and gradual cooperative adaptation

Overall, the governance complex that arose from Chicago was strongly decentralized and lacking in synergy, giving rise to demand for some form of management. Given sharply conflicting state preferences, a general top-down restructuring of the GGC was off the table (Hypothesis 2). Nevertheless, limited top-down adjustments were made. The move to a purely market-based system rendered redundant many existing intergovernmental aviation organizations. As no state saw an interest in continuing to fund these obsolete organizations doing away with them was relatively easy (Hypothesis 2). In May 1947 the International Technical Committee of Experts in Air Law was dissolved by member states, its functions transferred to ICAO along with those of the International Legal Committee on Aviation (Wilberforce 1947). The many regional and international aeronautical congresses and conferences also ceased to meet - their functions rendered irrelevant by the new Chicago regime.

As predicted, given limited top-down restructuring, bottom-up adaptation also took place (Hypothesis 3). Between the two overarching governance bodies—ICAO and IATA—an increasingly stable of division of labor emerged. While ICAO promoted multilateral cooperation to standardize navigation practices, IATA facilitated cooperation among airlines (many still state-owned) on scheduling, reservation and communication systems, handling of passengers, baggage, and cargo (Lissitzyn 1964). Despite frequent bilateral conflicts between states regarding the allocation of routes and capacities, significant task-differentiation meant that IATA and ICAO experienced few negative spill-overs from such conflicts and could continue to consolidate their separate functions (Jönsson 1981:281; Nayar 1995:158).

Over time, bottom-up adaptation evolved beyond task-differentiation to involve growing cooperation (Hypothesis 4). The consolidation of a system of “free but controlled competition” (Jönsson 1981; Goodwin 1964) ushered in a highly competitive environment both for airlines and for the many public and private institutions whose technical guidelines remained voluntary. Growing institutional density combined with slower market growth meant fewer resources for institutions to draw on. The gradual response was to form regional alliances and industry bodies in an effort to strengthen bargaining positions. Between 1952 and 1973 no less than seven regional airline organizations were formed representing state-owned and private airlines (Jönsson 1981:284). Other institutional agents also adapted to competition by pooling resources. In 1970, the three major airport associations (Airport Operators Council International; International Civil Airports Association; and Western European Airports Association) merged to form the Airports Associations Coordinating Council to defend their joint interests. Similar industry alliances formed among air-carriers and pilots (e.g., the International Federation of Airline Pilots Associations and International Air Carrier Association both created in 1971). Thus, consistent with Hypothesis 4, growing resource-scarcity at the system level led to growing resource-pooling among component institutions.

### 1973–1990: Successive market shocks and growing disorder

During the 1960s and 1970s a series of shocks hit the global airline industry. First, the birth of jet airliners and the introduction of national carriers in many developing countries led to a rapid expansion of transport capacity which far outstripped consumer demand (US Government 1963). In an increasingly globalized market, both US and British market shares declined rapidly (Lissitzyn 1964; Jönsson 1981). But while London responded to growing competition by continuing to push for centralized regulation of international routes and fares, Washington sought further deregulation in the hope of gaining a competitive edge. “The size of the US aviation market tends to give our aviation policies much weight in the world air transport system. This influence must be placed on the side of expansion” a US Government statement concluded. “Any policy or arbitrarily restricting capacity, dividing markets by carrier agreements, encouraging high rates or curtailing service for which a demand exists, would be harmful to our national interests” (US Government 1963; Goodwin 1964).

The oil shocks of 1973 and 1975, which increased airline operating costs, further widened the gulf between British and American preferences (Jönsson 1981:287). In 1976 Britain denounced the bilateral agreement governing US-UK commercial routes, demanding an equal share of transatlantic traffic, a larger number of gateways into the US, and a more effective system for regulating capacity. Washington responded by threatening to rescind IATA’s exemption from US antitrust legislation, thus barring foreign airlines from operating in the US with collectively pre-determined fares. Despite objections from other states to this “extraterritorial application” of US antitrust law (Jönsson 1981:288), IATA’s central fare-setting role was undermined and “open pricing” became the norm on international routes. With this, intergovernmental cooperation was effectively reduced to voluntary coordination of technical standards through ICAO (along with a number of conventions against terrorism-related acts involving aircraft). Alongside ICAO private industry organizations representing air carriers, airports, cargo-operators, etc. defended their separate “national” commercial interests in a hyper-competitive global market. The result was a largely conflictual, disordered system.

### 1990s to the present: Widening of the GGC and growing collaboration

The predominantly market-based system for regulating international civil aviation that took shape in the 1970s might seem a natural endpoint given the preponderance of powerful private commercial actors in this domain. Still the relative lack of centralized coordination is far from a foregone conclusion, as the evolution of the GGC since 1910 demonstrates. Until the 1960s most national airlines were government-owned and operated. Even private airlines had the status of “flag-carriers”, heavily funded and monitored by states. Hence, there is nothing “inevitable” about the lack of centralized cooperation in this area. Rather, a decentralized, fragmented system reflects repeated failures of top-down restructuring of the governance complex, which have been only partly compensated for by bottom-up adaptation.

Since the 1990s, new challenges from international terrorism and global health emergencies have led to increasing overlap between the GGC for civil aviation and adjacent governance complexes, leading to an expansion of existing governance agendas (see Table 3). Rather than leading to growing disorder, however, new challenges have largely been addressed through decentralized coordination and collaboration. 9/11 shook the global aviation industry, ushering in stricter airport regulations and government-mandated changes to commercial aircraft. However, due to ICAO’s limited authority, beyond certain minimum requirements, safety standards and procedures vary significant from country to county.^17^ In short, governance of aviation safety remains decentralized and fragmented. Nevertheless, regular briefings and consultations between the UN Security Council Counter-Terrorism Committee and ICAO focus on promoting worldwide adoption of more exacting universal standards.^18^

**Table 3:**
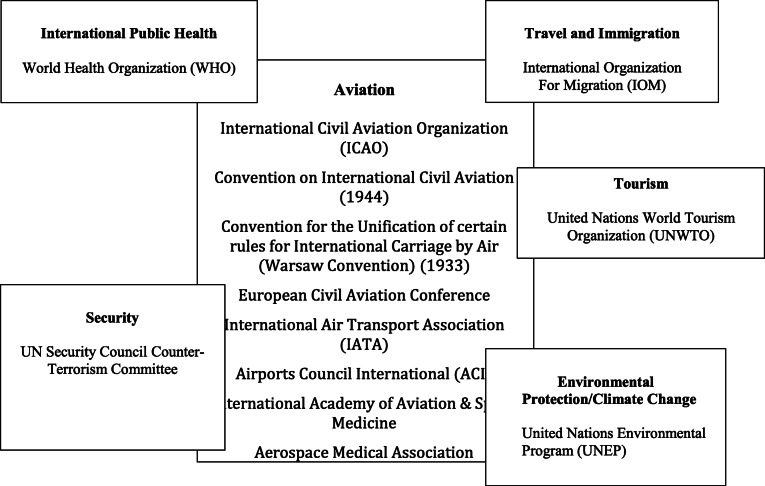
The International Civil Aviation Complex, 1910-present: Major IOs addressing issues related to international aviation

More recently, public health emergencies such as SARS, Ebola, and SARS-CoV-2 (Covid-19) have created potential for regulatory conflict by placing air travel within the scope of two overlapping domains of international law: the Chicago Convention, and the WHO’s International Health Regulations (IHRs) (ÓCuinn and Switzer 2019). Article 14 of the Chicago Convention obliges contracting states to “take effective measures to prevent the spread by means of air navigation of…communicable diseases”. The WHO’s IHRs aim “to prevent, protect against, control and provide a public health response to the international spread of disease in ways…which avoid unnecessary interference with international traffic and trade” (IHR 2005). The potential for jurisdictional conflict seems clear.

So far, ICAO and the WHO have sought to adapt to new challenges through coordination and collaboration. Since the 1990s ICAO has been involved in international consultations to revise the WHO’s existing IHRs in light of the return of old epidemics like cholera in some parts of the world, and the emergence of new infectious diseases like Ebola.^19^ In addition to consultation on policy-development, the two organizations have collaborated on practical issues such as quarantine, disinsectization of aircraft, and airport health and sanitary facilities (ÓCuinn and Switzer 2019). In light of growing risks of transmitting infectious agents via air-travel, ICAO in 2004 reviewed the compatibility of its aviation standards with emerging new standards for international public health and concluded that, as a result of ongoing consultations, the Chicago Convention and WHO’s IHR were “generally synergistic”.^20^

Regulatory overlap has also occasionally led to conflict. The 2003 SARS pandemic damaged consumer confidence in the airline industry, leading airlines to cut flights to affected areas, mainly in the Asia-Pacific.^21^ Meanwhile, the WHO counselled *against* flight cancellations on grounds that travel restrictions would have dire economic consequences for affected countries while doing little to stop the spread of disease (ÓCuinn and Switzer 2019). During the 2014 Ebola outbreak in West Africa, ICAO again instituted mass cancellation of flights to affected countries against WHO advice.^22^ Mostly, however, ICAO and IATA have chosen to defer to the WHO’s authority. In the aftermath of SARS, ICAO enacted a series of updates to its Standards and Recommended Practices (SARPS) under the Chicago Convention, carefully cross-referencing the WHO’s IHRs. After the 2014 Ebola-outbreak trilateral consultations between ICAO, IATA and the WHO led to the formulation of emergency measures which allowed normal air-travel to resume (ÓCuinn and Switzer 2019:77).

With time, adaptation has evolved from informal coordination to institutionalized collaboration (Hypothesis 4). In 2006 representatives from ICAO, the WHO, IATA, the Airports Council International and the UN World Tourism Organization founded the Collaborative Arrangement for the Prevention and Management of Public Health Events in Civil Aviation (CAPSCA) to keep emergency guidelines for states up to date. Focused on facilitating safe air-transport during global health emergencies, CAPSCA “performs the important function of ‘overlap manager’ between different domains of law” (ÓCuinn and Switzer 2019:78–9).^23^ The potential for regulatory conflict has thus been gradually reduced, first through coordination, and eventually by affected institutions pooling forces to confront joint challenges.

### Case summary

The GGC for civil aviation has evolved through stages characterized by varying levels of (dis)order. During a first stage (1900–1943) rapid technological developments combined with conflicting state interests triggered rapid proliferation of overlapping institutions. During this period, a resource-rich environment facilitated a combination of individual task-expansion and informal decentralized coordination. Despite growing institutional density, the system thus remained relatively ordered as separate institutions supplied overlapping but compatible functions. During a second period (1944–72) growing institutional fragmentation prompted limited top-down restructuring, followed by gradual bottom-up adaptation through growing task-differentiation and resource-pooling. A third stage (1973–1990) was marked by severe economic shocks to the aviation industry, leading to increasing market competition and growing disorder in respect to the governance of international routes, frequencies and fares. Since the 1990s, growing overlap between the functions of ICAO and IATA and institutions in adjacent governance domains like public health and security has been addressed mainly through bottom-up adaptation, ensuring broadly synergistic policy outcomes.

Overall, the evolution of the GGC illustrates that institutional density and disorder often increase in the wake of exogenous shocks which upset existing exchange relationship. Between shocks, however, state principals have (sometimes) succeeded in rationalizing cooperation by terminating or reforming redundant institutions, while institutional agents have sought to improve their individual fitness through coordination and/or resource-exchange thereby providing an element of bottom-up ordering of the system as a whole. The GGC has thus evolved through a pattern of punctuated equilibrium in which periodic shocks have resulted in growing disorder, followed by periods of (re)consolidation via top-down restructuring and bottom-up adaptation (Hypothesis 5).

## Conclusions

Many observers have argued that a proliferation of international agreements and international law in recent decades is causing a deleterious fragmentation of global institutional architectures. There can be little doubt that the international system has become increasingly “crowded” in terms of the sheer number of institutions in play (Alter and Raustiala 2018). Nevertheless, institutional overlap and legal fragmentation are not a recent phenomenon. As this article has illustrated, the schism between integration v. fragmentation of governance functions lies at the heart of the international institutional system as it has evolved since the early twentieth century.

This article has focused on how states and other political agents adjust to growing institutional complexity. I have identified important drivers of growing institutional overlap and disorder, such as exogenous shocks and competing political agendas which impede states’ efforts to limit or control institutional proliferation and promote reliance on informal mechanisms to manage complexity in lieu of institutional re-integration (see also Fioretos 2020). I have also identified conditions that facilitate “ordering” of governance complexes, including congruent state preferences and an abundance of institutional resources (such as demand for regulation, availability of private and public funding and expert staff) that enable bottom-up adaptation. While there is nothing inevitable or lawlike about the processes of top-down and bottom-up ordering I have identified, my analysis shows that institutional proliferation is not a unidirectional process leading to ever-increasing fragmentation and disorder, but that the opposite process can also be expected and be frequently observed: fragmented and disordered governance systems growing increasingly ordered over time.

The findings of this study have theoretical implications beyond the case of international aviation. In theoretical terms, my analysis attests to the significance of international bureaucracies as important agents of institutional stability and change. In many instances, inability by state principals to integrate fragmented governance structures in the aviation domain have been compensated for through decentralized adjustments by both public and private governance agents.

My analysis also highlights the path-dependent nature of processes of institutional change. The failure to establish a single international focal regime to govern civil aviation at the beginning of the twentieth century prompted a rapid proliferation of separate governance bodies and institutionalized a geographic divide in international governance which made it more difficult, subsequently, for states to reduce institutional complexity by adding new agenda-items to existing institutional agendas, or by merging or subordinating new institutions to existing ones. The fact that recent exogenous shocks to the GGC, such as new forms of international terrorism and global epidemics, have been accommodated through bottom-up adaptation rather than top-down adjustment can also be understood (in part) as the result of weak intergovernmental governance mechanisms in this domain which have their origin in the early post-war period. The history of international aviation governance thus vividly illustrates how the emergence of new rules and practices is shaped and constrained by existing institutional frameworks (Raustiala and Victor 2004:279–80; Hofmann 2009, 2013). At the same time, however, the case narrative also points to the importance of “critical junctures” (Fioretos 2017) such as the 1944 Chicago Conference which open possibilities for multiple institutions to change in tandem, thereby setting a governance system on a different evolutionary path.

This article has provided a detailed analysis of the evolution of the GGC for civil aviation in order to trace how this specific governance domain has fluctuated between order and disorder over time. Similar dynamics may be observed in other policy domains. Thus, an important avenue for future research is to widen the empirical basis for studying dynamics of top-down and bottom-up ordering of institutional relationships through systematic comparison to other GGCs.

At the same time as it invites comparison between different domains of global governance, my analysis also highlights the promise of studying the evolution of *single* GGCs across time. Many observers have linked growing institutional complexity to the end of the Cold War (Alter and Raustiala 2018: 212; Kahler 2020). Although they are rarely explicitly articulated, one can identify (at least) three broad explanations for the apparent increase in institutional complexity at this historical moment. First, some argue that a global power shift away from the main founders and chief beneficiaries of major postwar international institutions has led to growing institutional deadlock and has encouraged the creation of competing venues for cooperation. Second, rapid technological progress has made it easier to organize transnationally thereby encouraging the creation of new governance schemes to advance previously underrepresented interests. Third, neoliberalism has led to the emergence of new private governance agents and initiatives alongside states and IGOs. These systemic factors are all plausible drivers of growing institutional diversity, density and overlap. But while they may explain a *general* increase in institutional complexity since the end of the Cold War, these macro-level factors are too indiscriminate to account for variation across different policy domains or within specific domains over time. Indeed, critical junctures at which choices become decisive for the selection of one institutional path over other possible options are often limited to a single policy domain (Hall 2016), leading to different institutional trajectories. Exploring variation in the historical evolution of GGCs may thus help us to distinguish between *systemic* causes of growing complexity and *proximate* causes that lead to institutional proliferation in specific instances.

## Footnotes

1 The leading study is Jönsson (1981) on which I draw in Section 3. Richards (2001) looks more narrowly at the post-WWII ‘Bermuda Agreement’ on capacity allocation. Beyond these studies international civil aviation governance is (to my knowledge) relatively unexplored by international relations scholars.

2 First Standing Committee on Delegated Legislation, 13 December 2004: Draft Agency for International Trade Information and Co-operation (Legal Capacities) Order 2004.

3 A 2007 report from the Danish Ministry of Foreign Affairs complained that AITIC competed for donor resources with other similar bodies.

4 http://www.wahooas.org/spip.php?page=rubriqueS&id_rubrique=24&lang=en.

5 At the League’s founding many existing IOs were absorbed into its legal structure. Archives of the League of Nations; Reinalda and Kille 2017:225.

6 An illustrative example is the UN Relief and Rehabilitation Administration (UNRA). After it was dissolved in 1947 UNRA’s functions were reallocated among the International Refugee Organization, the WHO, the FAO and UNICEF (Wessel 2011:347).

7 Ftn.2.

8 This is, of course, merely the reverse logic of the widespread notion that clashing state interests is what triggers institutional proliferation in the first place.

9 Relevant resources can be conceived as demand for governance and availability of funding and expert staff.

10 Art.34, Convention Relating to the Regulation of Aerial Navigation, Paris, 13 October 1919.

11 Although the first transatlantic flight took place in 1919, the first *passenger* trip was accomplished in 1927 and scheduled transatlantic passenger flights began in the 1930s. Civil aviation in the USSR remained a purely domestic matter until Aeroflot introduced its first international route between Moscow and Prague in 1936.

12 https://applications.icao.int/postalhistory/international_aviation_organizations_working_alongside_ican_part_1.htm. Assessed 25-09-2020.

13 Postal History of ICAO.

14 While European Allies focused their wartime production on fighter planes and bombers, the US specialized in transport aircraft, thereby expanding its capacity for producing planes for civilian use.

15 ‘Churchill and Roosevelt: The Complete Correspondence,’ edited by Warren F. Kimball, (Princeton 1984), vol. 3:407.

16 UN Treaty Series, vol.15, 1948:296.

17 https://www.cfr.org/backgrounder/targets-terrorists-post-911-aviation-security

18 https://www.un.org/sc/ctc/news/keyword/aviation-security/

19 https://www.who.int/ihr/revisionprocess/revision/en/. (Assessed 25-09-2020).

20 ICAO 2004: A35–12: Protection of the health of passengers and crews and prevention of the spread of communicable disease through international travel.

21 https://www.iata.org/en/iata-repository/publications/economic-reports/what-can-we-learn-from-past-pandemic-episodes/ (Assessed 25-09-2020).

22 www.who.int/mediacentre/news/statements/2014/ebola-2nd-ihr-meeting/en/(Assessed 25-09-2020).

23 www.capsca.org/IntroandObjectivesWeb.pdf (Assessed 25-09-2020).
